# Complete Endoscopic and Histopathological Remission of Mantle Cell Lymphoma of the Gastrointestinal Tract

**DOI:** 10.7759/cureus.4350

**Published:** 2019-03-31

**Authors:** Nabeeha Mohy-ud-din, Aritra Guha, Marcia Mitre

**Affiliations:** 1 Internal Medicine, Allegheny Health Network, Pittsburgh, USA; 2 Gastroenterology, Allegheny Health Network, Pittsburgh, USA

**Keywords:** endoscopy, mantle cell lymphoma

## Abstract

Mantle cell lymphoma (MCL) is a rare and incurable subtype of non-Hodgkin’s lymphoma (NHL). Primary gastrointestinal (GI) MCLs are even rarer, accounting for only 1%-4% of GI lymphomas. We describe a case of a 77-year-old female who presented with complaints of indigestion and abdominal bloating. An upper endoscopy was performed which revealed a duodenal bulb polyp, biopsies of which were consistent with MCL. She was initially observed without any chemotherapy; however, a repeat endoscopy two years later revealed that she now also had MCL of the ileocecal valve. The patient was initiated on treatment with rituximab, cyclophosphamide, vincristine, and prednisone (R-CVP). She underwent regular surveillance with her oncologist after completion of her chemotherapy and repeat surveillance scans remained negative for any recurrence. A repeat upper endoscopy with endoscopic ultrasound and colonoscopy were performed which showed complete endoscopic and histopathological remission of her lymphoma. Patients with MCL typically have a poor prognosis; however, our patient remains symptom free and in complete remission six years from her initial diagnosis.

## Introduction

Mantle cell lymphoma (MCL) is a rare and incurable subtype of non-Hodgkin's lymphoma (NHL) that often behaves aggressively. Patients often present with advanced stage disease with a median survival of three to five years [1]. Primary gastrointestinal (GI) MCL is very rare, accounting for only 1%-4% of GI lymphomas [2]. Macroscopic GI involvement in MCL is rare (15%-30% of patients) and there are sparse reports of MCL detected by endoscopy [3-4]. We describe an unusual presentation of MCL presenting as a duodenal mass and then also occurring in the ileocecal region, achieving complete endoscopic and histological remission after chemoimmunotherapy.

## Case presentation

A 77-year-old female who had previously been treated for *Helicobacter pylori* gastritis, presented with a six-month history of indigestion, heartburn, and abdominal bloating in 2012. An upper GI endoscopy was performed which revealed a duodenal bulb polyp. Biopsy of the duodenal polyp revealed a clonal population of malignant B-cells with a CD5+, CD10-, CD20+, CD23- immunophenotype. There was also over-expression of cyclin D1 consistent with a diagnosis of MCL.

The patient underwent a positron emission tomography/computed tomography (PET/CT) scan for staging which showed an enlarged inguinal lymph node and a nonenlarged left external iliac lymph node. There was no bone or central nervous system involvement and her bone marrow biopsy was normal. Because of the indolent nature of her presentation, observation alone was recommended and the patient was followed closely with regular clinic visits, monthly labs including a complete blood count (CBC) and lactate dehydrogenase (LDH) levels, as well as surveillance PET/CTs every three months. 

**Figure 1:**
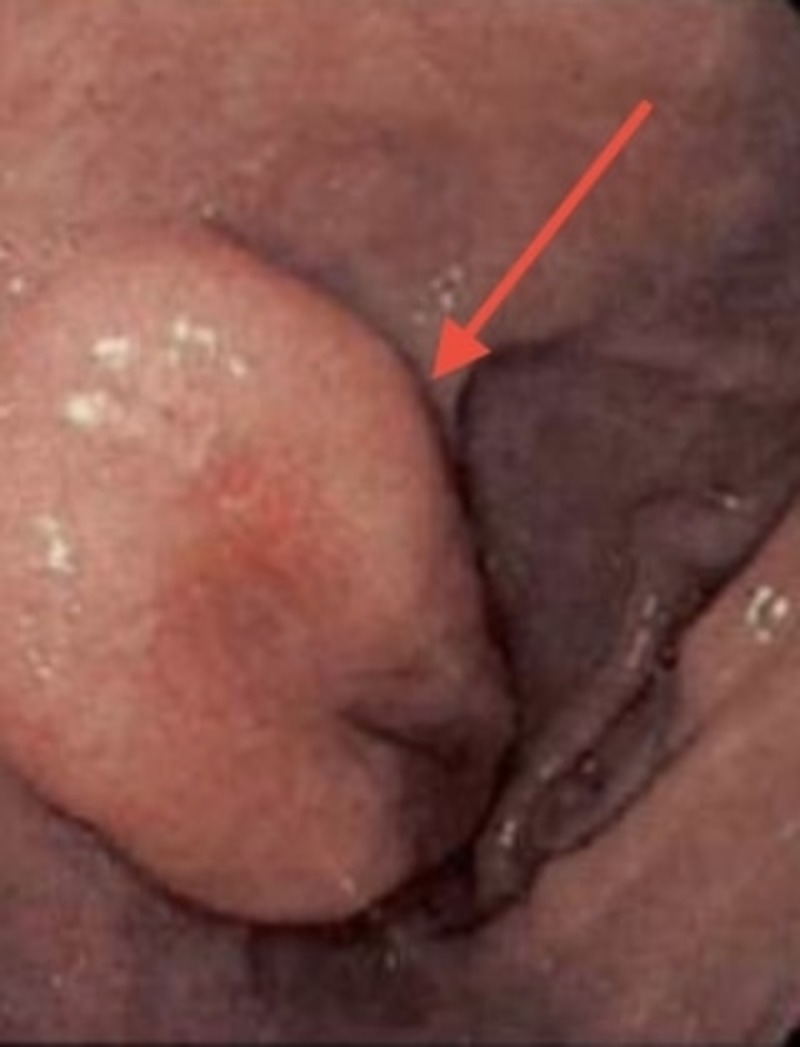
Endoscopically visible lesion in the duodenal bulb.

In November 2014, the patient underwent a repeat upper endoscopy with endosonographic ultrasound and colonoscopy for surveillance which revealed an increase in the size of the duodenal bulb lesion as shown in Figure 1. 

**Figure 2:**
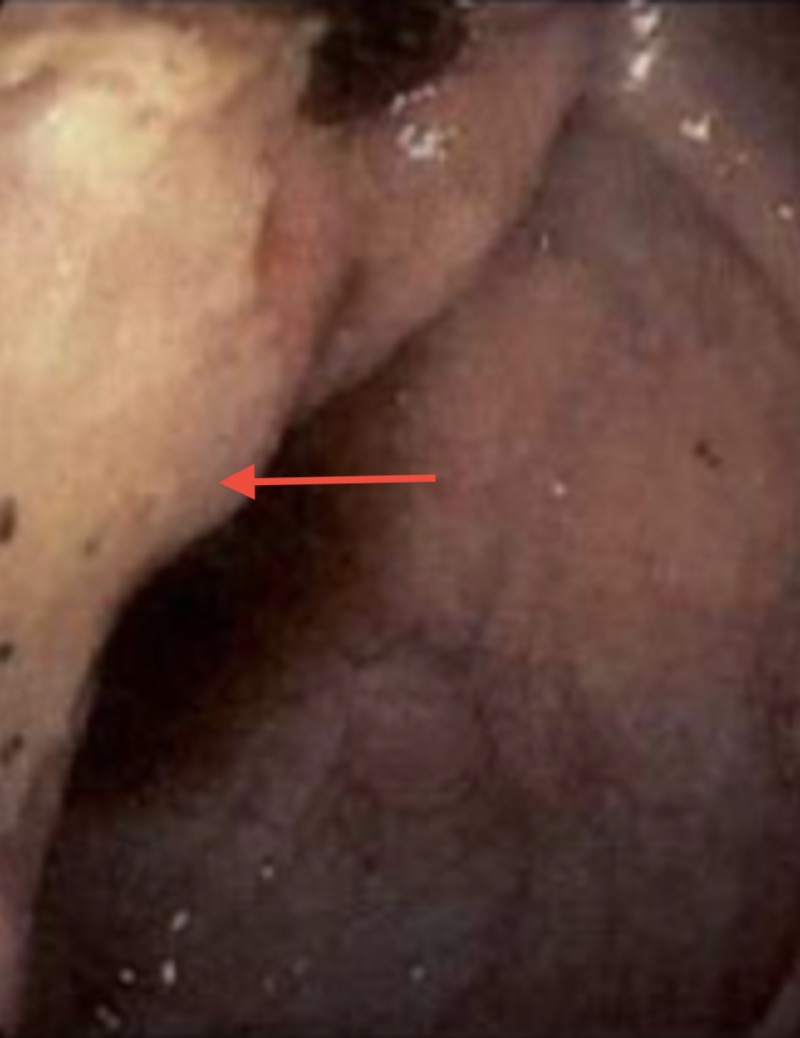
Endoscopic view revealing ulcerated mucosa and edema at the ileocecal valve.

She was also noted to have abnormal mucosa in the ileocecal valve as illustrated in Figure 2. 

**Figure 3:**
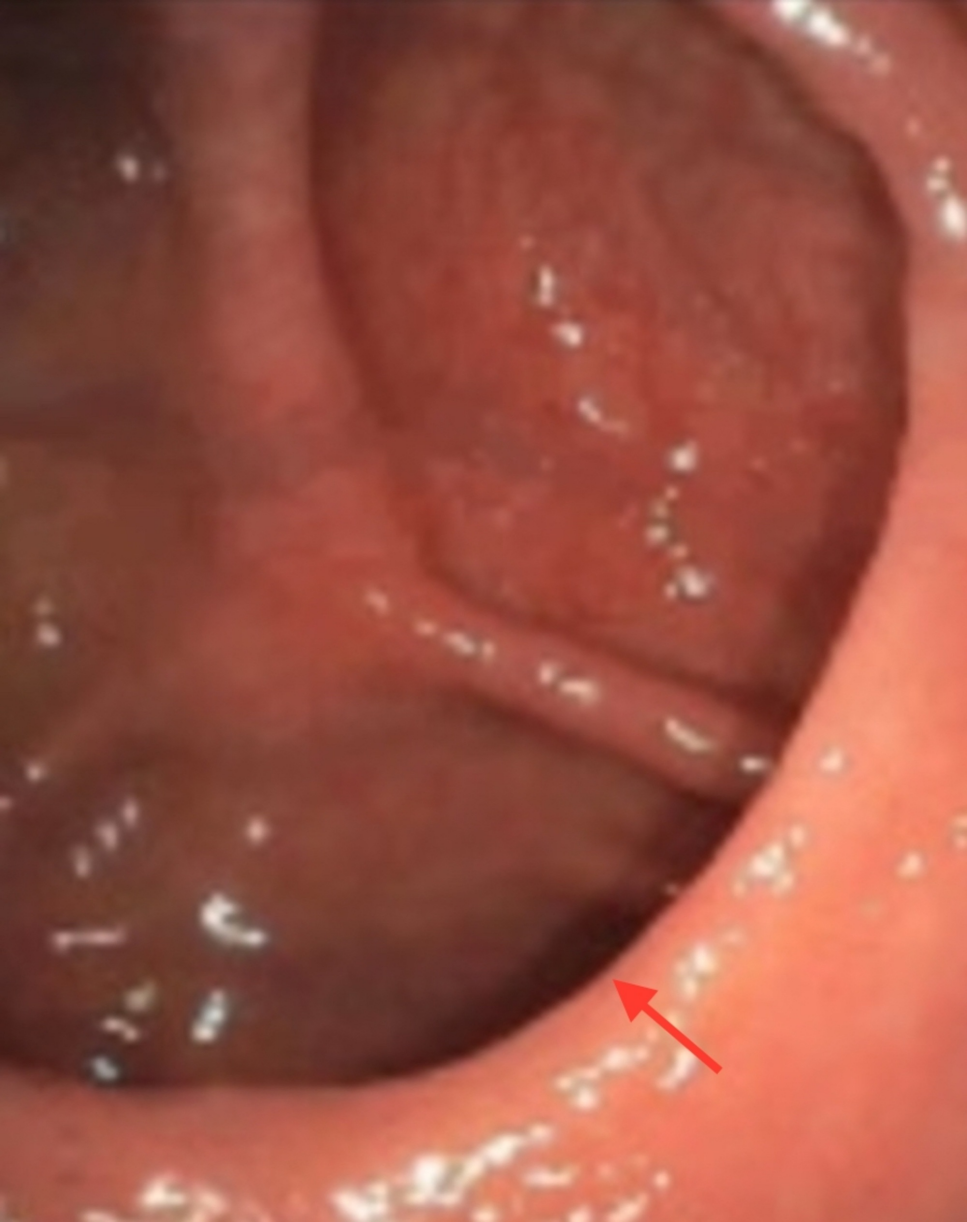
Normal endoscopic appearance of the ileocecal valve.

Biopsy of the ileocecal valve revealed residual MCL. The patient was initiated on treatment with rituximab, cyclophosphamide, vincristine, and prednisone (R-CVP) and received a total of six cycles in 2015. She underwent regular surveillance with her oncologist and was noted to have no recurrence of her disease on repeat PET/CT scans. A repeat upper endoscopy with endoscopic ultrasound in December 2018 revealed normal appearance of the duodenal bulb. A repeat colonoscopy was also performed which revealed normal endoscopic appearance of the ileocecal valve as shown in Figure 3. 

## Discussion

Mantle cell lymphoma is a rare subtype of NHL with a poor prognosis [1]. MCL can present in the GI tract as multiple masses, nodules, ulcers, polyps, or thickening of the intestinal walls [4]. The most common GI site of lymphomatous involvement is the stomach followed by the ileocecal region [5].

The most common presenting symptoms of GI MCL are abdominal pain, diarrhea, and hematochezia. It is diagnosed by endoscopy, histopathology of tissue samples, immunohistochemistry, and cytogenetic studies. The most common endoscopic appearance is lymphomatous polyposis and it rarely presents as protuberant or superficial lesions [6].

Chung et al. described seven cases of MCLs of the GI tract in a six-year period. Six out of seven of these cases showed multiple polyposis and all of these occurred in the small bowel and colon [7]. The most common frontline treatment for MCL is combination chemoimmunotherapy with cyclophosphamide, adriamycin, vincristine, and prednisone plus rituximab (R-CHOP) or bendamustine and rituximab (BR) [8]. The median overall survival with conventional chemotherapy ranges from three to five years [1]. A recent study showed that there was no statistically significant difference in overall survival in patients with MCL who have GI involvement compared to patients who do not [9].

Our case presents a rare occurrence of endoscopically detectable MCL which achieved complete endoscopic and histopathological remission after chemoimmunotherapy. Our patient is now six years from her initial diagnosis and remains in complete remission. 

## Conclusions

This is an unusual and rare case of complete histopathological and endoscopic remission of primary GI lymphoma, which is historically a disease with a very poor prognosis.
